# Supplementary material for: Stress-induced artificial neuron spiking in diffusive memristors
Source: Commun Eng. 2024 Nov 9;3:163. doi: 10.1038/s44172-024-00315-z (PMC11550850; doi:10.1038/s44172-024-00315-z)
Supplement: Supplementary file 2 — Supplemnetary Information [file 44172_2024_315_MOESM2_ESM.pdf]

Supplementary information for Stress-induced artificial neuron spiking in diffusive memristors.

Supplementary Note1: IN order to confirm, that our memristor device does not produce spikes at impact pressure less than 0.1MPa, Fog. S1 shows measured voltage for impact 0.05MPa.

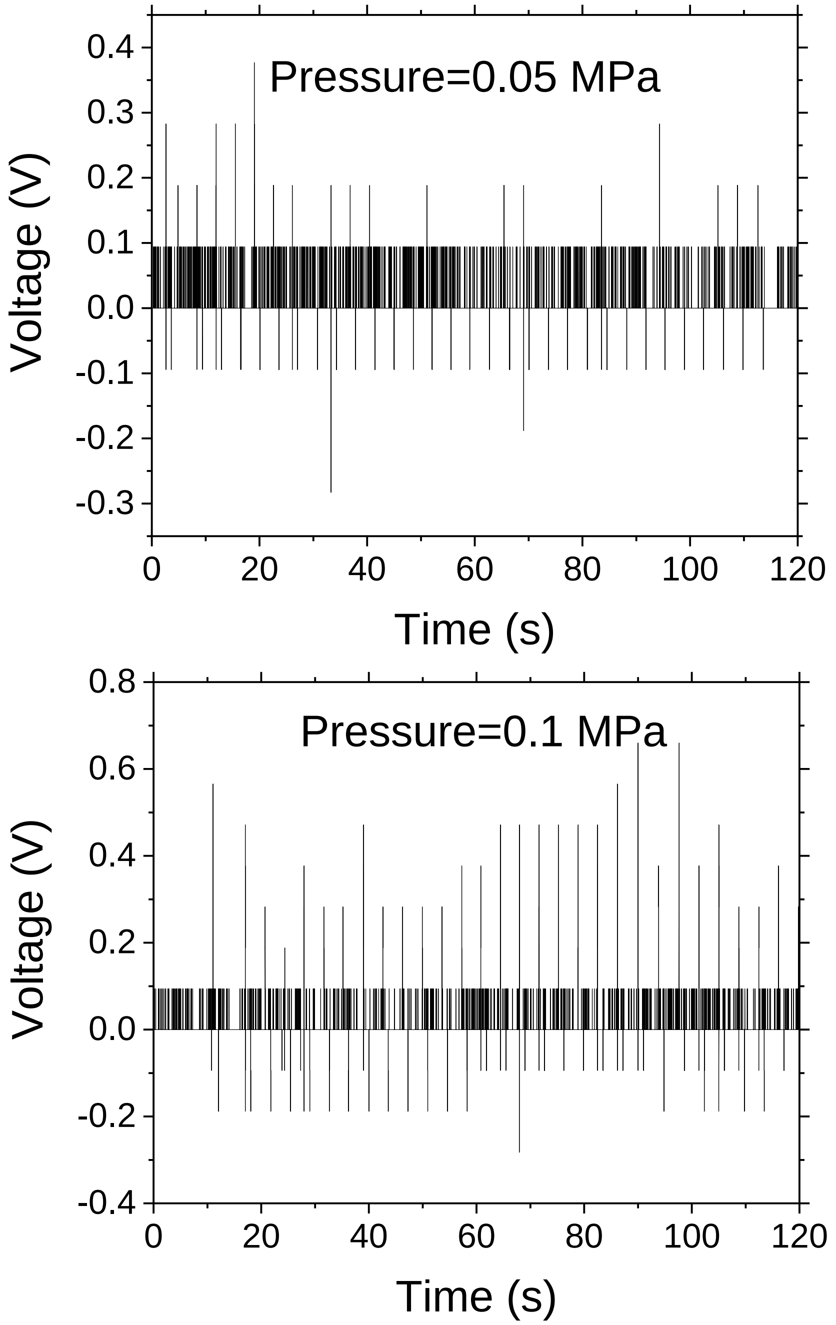

Fig. Supplementary Note 1. Measured voltage on the memristor for impact pressure 0.05 MPa, and 0.1 MPa with 0.2 s time interval between subsequent impacts. The measured voltage does not demonstrate spiking behaviour for low pressure values.

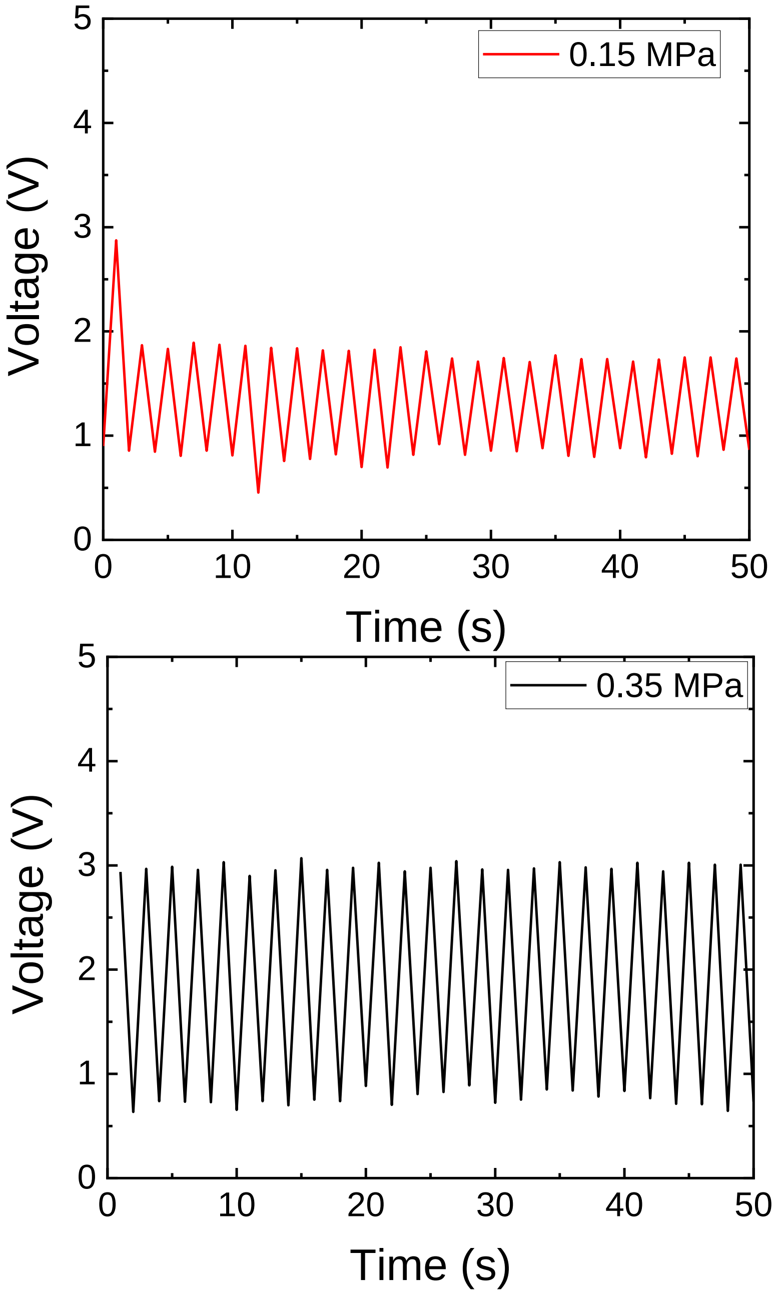

Fig. Supplementary Note 2. Measured voltage for a commercial force sensor at impact pressure 0.15 MPa and 0.35MPa

Supplementary Note 3: Equation used for the model:

$$\frac{d\tilde{x}_i}{d\tilde{t}} = -(1 - \Delta_U f(\tilde{t}))\frac{\partial \tilde{U}(\tilde{x}_i)}{\partial \tilde{x}_i} + 0.633\tilde{V} + \sqrt{2\tilde{T}}\,\xi_i(\tilde{t}), \qquad \text{(S1a)}$$

$$\frac{d\tilde{T}}{d\tilde{t}} = 0.18\frac{\tilde{V}^2}{\tilde{r}(x_1,\dots x_N)} - \tilde{\kappa}\,\tilde{T}, \qquad \text{(S1b)}$$

$$18\left(1 + \left(\frac{C_2}{C_1} - 1\right)f(\tilde{t})\right)\frac{d\tilde{V}}{d\tilde{t}} = \tilde{V}_{ext} - \left(1 + \frac{\tilde{R}_{ext}}{\tilde{r}(\tilde{x}_1,\dots\tilde{x}_N)}\right)\tilde{V}, \qquad \text{(S3c)}$$

where $\tilde{r} = \cosh(\tilde{x}_1/0.12)$.

Here, we normalise time as $t = \tau\,\tilde{t}/18$, Ag-cluster position as $x = (L/2)\tilde{x}$ with L being the size of the gap/bottleneck, the bottleneck resistance R and external resistance R_{ext} are normalised by the minimal resistance of the bottleneck, Ag cluster temperature is normalised as $T = (2.25\eta L^2/\tau k_B)\tilde{T}$, the voltage across the bottleneck is normalised as $V = (14.2\,\eta L^2/q\tau)\tilde{V}$, electrochemical potential is normalised as $U = 4.5\,\eta L^2\tilde{U}/\tau$ and is provided in the uploaded data set, the electron tunnelling length $\lambda = (L/2)\tilde{\lambda}$ was set to be 0.12, the cooling constant normalised as $\tilde{\kappa} = \kappa\tau/18$ was set to be 0.9, and thermal capacitance was set to be $C_{th} = 27.7\eta L^2 k_B/q^2 R_0$. The periodic impact application occurs with period t_p/τ varying from 0.05 to 2. We also assume that the bath temperature is negligible ($\tilde{T}_0 \approx 0$) comparing with the bottleneck cluster temperature where all heat is generated.

This is a compact phenomenological model, there the complex dynamics of Ag-clusters forming filaments is reduced to a one Ag cluster (a particle) model. Such one particle approximation has been justified by extensive simulations in [1] showing that the resistance is well described by dynamics of the particle in almost formed conductive filament.

The dynamic of the cluster shuttling in the bottleneck is overdamped and controlled by the electrochemical potential landscape, the electric force generated by voltage applied to the memristor terminal, and diffusion random forces. The heat dynamics is reduced to the Newton model, where the heat diffusion is inside the memristor device reduced to an ordinary differential equation describing heat removal to the bath. This model is again consistent with the view that all heat is produced by local Joule dissipation in the bottleneck. Away from the bottleneck, the conductive filament is widening resulting in much easier heat removal. Therefore, the heat resistance is determined by the region near the bottleneck and can be reduce to a single constant. The potential $\tilde{U}$ has a single minimum at $\tilde{x} = -1$ and shown below.

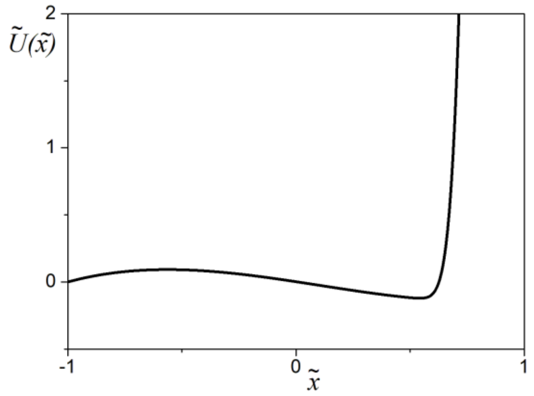

Fig Supplementary Note 4: Potential used for stochastic simulations

Supplementary Note 5. Estimation of the dielectric constant modification due to the elastic strain:

According to [2] the relative change $\Delta\varepsilon$ in the absolute dielectric constant because of an elastic stress σ is:

$$\Delta\varepsilon = 2M_{ij}\sigma$$

where M_{ij} is the corresponding dielectrostriction coefficient. According to Yimnirun et al. [3] for fused silica $M_{11} \approx M_{12}$= -0.2x10^{-21} m^2/V^2.

We consider some part of the silica film that is grown on a wrinkled PET film as being bent in an ideal arc form with the corresponding radius of curvature *R* before the impact. Upon the impact the assumption is this kind of an arc disappears, and the film becomes flat. The neutral plane of a SiO_2 film is assumed to lay in the middle of the film thickness *d*, at a distance *d*/2 from the film surface. Under a crude approximation of the film plane as a beam, when the film is bent in an arc the corresponding longitudinal stress in a film plane at a distance *y* from the neutral plane is $\sigma(y) = E\frac{y}{R}$, [4] where E is the Young's modules, for SiO_2 E=73 GPa according to [1].

We identified some arcs in the wrinkled film cross section by taking magnified optical camera images and fitting to a circular arc. The smallest radius of curvature was found to be *R*=0.150 mm. By taking the maximal distance from the neutral plane y_{max}=*d*/2=50 nm, and substituting the above values for *E* and *M* we obtain $\Delta\varepsilon = 0.001\varepsilon_0$ which amounts to ~ 0.03% relative effect if for SiO2 $\varepsilon \approx 4\varepsilon_0$.

Supplementary References:

[1] Yi, Wei, et al. "Quantized conductance coincides with state instability and excess noise in tantalum oxide memristors." *Nature communications* 7.1 (2016): 11142.

[2] Huang, J.-Q., Huang, Q.-A., Qin, M., Dong, W., Chen, X.: Strain effect of the dielectric constant in silicon dioxide. Journal of microelectromechanical systems 19(6), 1521–1523 (2010)

[3] Yimnirun, R., Moses, P.J., Newnham, R.E. *et al.* Electrostrictive Strain in Low-Permittivity Dielectrics. *Journal of Electroceramics* **8**, 87–98 (2002).

[4] Engineering Mechanics 2, Mechanics of Materials, by D. Gross et al., Springer Berlin, Heidelberg (2018)
